# Genetic Diversity of Circumsporozoite Surface Protein of *Plasmodium vivax* from the Central Highlands, Vietnam

**DOI:** 10.3390/pathogens11101158

**Published:** 2022-10-07

**Authors:** Tuấn Cường Võ, Nguyen Thi Minh Trinh, Hương Giang Lê, Jung-Mi Kang, Won Gi Yoo, Huynh Hong Quang, Byoung-Kuk Na

**Affiliations:** 1 Department of Parasitology and Tropical Medicine, Institute of Health Sciences, Gyeongsang National University College of Medicine, Jinju 52727, Korea; 2 Department of Convergence Medical Science, Gyeongsang National University, Jinju 52727, Korea; 3 Tropical Diseases Clinical and Treatment Research Department, Institute of Malariology, Parasitology, and Entomology Quy Nhon, Quy Nhon 590000, Vietnam

**Keywords:** *Plasmodium vivax*, Vietnam, circumsporozoite surface protein, genetic polymorphism, natural selection

## Abstract

The circumsporozoite surface protein of *Plasmodium vivax* (PvCSP) plays a critical role in parasite biology. It has been extensively studied as a leading vivax-malaria-vaccine candidate. In this study, the genetic polymorphism and natural selection of *pvcsp* in *P. vivax* isolates collected from the Central Highlands, Vietnam were analyzed to understand the genetic structure of the parasite circulating in the endemic area and to provide baseline information for effective vaccine development based on the protein. Only two major alleles, VK210 and VK247, were detected in Vietnamese *pvcsp,* with VK247 being the predominant one. The N-terminal and C-terminal regions of Vietnamese VK210 and VK247 variants showed a low genetic diversity. Amino acid substitutions, insertions of a single amino acid or octapeptide (ANKKAEDA in VK210 and ANKKAGDA in VK247), and tetrapeptide repeat motifs (GGNA) were the main factors generating genetic diversity in the two regions of the Vietnamese VK210 and VK247 variants. Interestingly, these two regions of Vietnamese *pvcsp* displayed a unique natural selection pressure distinct from global *pvcsp*, particularly with the neighboring Southeast Asian *pvcsp* population. Meanwhile, the central repeat region (CRR) in both the VK210 and VK247 variants showed a high degree of polymorphic characters, caused by varying numbers, types, and combinations of peptide repeat motifs (PRMs) in Vietnamese *pvcsp*. Highly complicated polymorphic patterns of the CRR were also detected in global *pvcsp*. These results expand our understanding of the genetic structure of Vietnamese *pvcsp* and the population dynamics of *P. vivax* in the Central Highlands, Vietnam.

## 1. Introduction

Malaria is one of the most important mosquito-borne infectious diseases globally. In humans, malaria is caused by five species of *Plasmodium,* including *P. falciparum*, *P. vivax*, *P. malariae*, *P. ovale*, and *P. knowlesi*, transmitted by *Anopheles* mosquitoes [1]. This infectious disease has been threatening human health since the beginning of human history [2]. Recently, global mortality and morbidity caused by malaria have decreased remarkably compared to the last few decades. These tremendous successes in global malaria prevention and control were achieved by the increased availability of insecticide-treated nets, indoor residual sprays, and better access to diagnosis and effective treatment using artemisinin-based therapies. However, malaria is still a global public health concern, with an estimated 241 million cases and more than 620,000 deaths in 2020 [3].

Among five human-infecting *Plasmodium* species, *P. vivax* shows the broadest geographical distribution. It is endemic in South Asia, Southeast Asia, and South America. Nevertheless, *P. vivax* has long been neglected as it causes a benign malaria with milder clinical manifestations and lower mortality than those caused by *P. falciparum*. However, *P. vivax* displays unique biological characteristics that challenge the control and elimination of vivax malaria. It is known that vivax malaria is more complicated and much harder to control than falciparum malaria. The liver stage form of the parasite, hypnozoites, can remain dormant for several weeks to months in human liver cells before it is reactivated [4]. The ability to continuously produce gametocytes, a high infectivity to mosquitoes, and a short development in the mosquito vector also contribute to the difficulty of controlling *P. vivax* [5]. *P. vivax* can also cause serious clinical manifestations, including death [6,7,8]. The emergence and spread of drug-resistant strains are adding weight to the burden of this benign parasite [9,10]. Therefore, developing an efficacious vaccine for vivax malaria is imperative.

The circumsporozoite protein (CSP) has been investigated extensively as one of the most attractive malaria vaccine candidates. This protein is a major antigen that covers the surface of *Plasmodium* sporozoites. It plays multiple critical roles, such as human hepatocyte invasion by parasites [11,12]. The gene encoding *P. vivax* CSP (PvCSP) has three different regions: a highly polymorphic and immunogenic central repeat region (CRR), flanked by two conserved non-repetitive N-terminal and C-terminal regions. The CRR of PvCSP is constructed with short polypeptide sequences, called peptide repeat motifs (PRMs), which repeat in tandem. PRMs can form unique motifs with different amino acid sequences, resulting in three distinct allelic variants: VK210, VK247, and *P. vivax*-like. VK210 and VK247 are the two most prevalent alleles that possess major nonapeptide repeat motifs, namely GDRA(A/D)GQPA and ANGA(G/D)(N/D)QPG, respectively [13,14]. Besides these variants, another variant motif of APGANQ(E/G)GGAA is also present in the CRR of *P. vivax*-like variants [15]. These motifs are usually repeated multiple times in the CRR. They contribute to high polymorphic characters of *pvcsp* along with synonymous and non-synonymous single nucleotide polymorphisms (SNPs) in the region [16,17].

Vivax malaria protein 001 (VMP001), one of the most advanced vivax-malaria-vaccine candidates currently, is a subunit vaccine based on PvCSP [18]. The Phase 1 trial of VMP001 has suggested that it could significantly delay parasitemia. However, it confers only a limited degree of protection against infection challenge [19]. Similar to other leading vaccine candidate antigens, the effectiveness of a PvCSP-based vaccine can be affected by genetic polymorphisms in the target region or protein. Therefore, understanding the genetic make-up of *pvcsp* in the wild-type *P. vivax* population would be informative for the development of a universal vaccine. Non-neglectable patterns of genetic polymorphism of *pvcsp* have been identified in global *P. vivax* isolates [17]. In this study, we investigated the genetic polymorphisms and natural selection of *pvcsp* from *P. vivax* isolates collected from the Central Highlands, a malaria-endemic region in Vietnam. A comparative analysis with global *pvcsp* was also performed to gain a deeper understanding of the genetic nature of *pvcsp* in the global population.

## 2. Materials and Methods

### 2.1. Blood Samples

Sixty-nine blood samples collected from *P. vivax-*infected patients were used in this study. The blood samples were obtained by finger pricks from malaria patients residing in four provinces (Dak Lak, Dak Nong, Gia Lai, and Kon Tum) in the Central Highlands, Vietnam, in 2018 and 2019 (Supplement File S1: Figure S1). Information on the blood samples and molecular diagnosis of the *P. vivax* infection were described in our previous report [20]. The Ministry of Health, Institutional Review Board of the Institute of Malariology, Parasitology and Entomology Quy Nhon, Vietnam (368/VSR-LSDT) reviewed and approved the study protocols, and informed consent was obtained from all participants before blood collection.

### 2.2. Amplification and Sequence Analysis of Vietnamese pvcsp

*P. vivax* genomic DNA was extracted from the dried blood spots using the QIAamp DNA Blood Kit (Qiagen, Hilden, Germany) according to the manufacturer’s protocols. The amplification of *pvcsp* in Vietnamese *P. vivax* isolates was performed by a nested polymerase chain reaction (PCR) with the same primer sets and amplification conditions as described previously [17]. To minimize nucleotide mismatching during the amplification steps, Ex Taq DNA polymerase (Takara, Otsu, Japan) with proofreading activity was used. Each PCR product was analyzed by electrophoresis on 1.5% agarose gel, extracted from the gel and cloned into a T&A cloning vector (Real Biotech Corporation, Banqiao City, Taiwan). Each ligation mixture was transformed into *Escherichia coli* DH5α competent cells. Positive clones with an appropriate insert were selected by colony PCR, and the nucleotide sequences of the cloned *pvcsp* were analyzed by automatic sequencing with M13 forward and M13 reverse primers. Plasmids from at least two individual clones from each isolate were sequenced to ensure sequence accuracy. The nucleotide sequences of Vietnamese *pvcsp* obtained in this study are accessible at the GenBank database under the accession numbers MW382969–MW383084.

### 2.3. Analyses of Genetic Diversity and Natural Selection of pvcsp

Nucleotide and deduced amino acid sequences of Vietnamese *pvcsp* were analyzed using Editseq and Seqman programs in the DNASTAR package (DNASTAR, Madison, WI, USA). The *pvcsp* sequences of Salvador I (Sal I; GU339059) and Papua New Guinea (PNG; M69059) strains were used as reference sequences to analyze the variants of Vietnamese *pvcsp* sequences. The values of the number of segregating sites (S), haplotypes (H), haplotype diversity (Hd), nucleotide diversity (π), and the average number of pair-wise nucleotide differences within a population (*K*) were estimated with DnaSP ver. 5.10.00 [21]. Values of synonymous (dS) and non-synonymous (dN) substitutions were analyzed and compared using the Z-test (*p* < 0.05 was considered statistically significant) with the MEGA6 program [22], using Nei and Gojobori’s method [23] with the Juke and Cantor correction. Based on the acquired values, the dN–dS value was calculated. Tajima’s D value [24] and Fu and Li’s D and F values [25] were calculated using DnaSP ver. 5.10.00 [21] to evaluate the neutral theory of natural selection.

### 2.4. Comparative Analyses of Genetic Diversity and Natural Selection in Global P. vivax Population

The genetic diversity and natural selection of *pvcsp* among the global *P. vivax* population were analyzed. The *pvcsp* sequences from *P. vivax* isolates (*n* = 632) from Myanmar (*n* = 171) [17], Cambodia (*n* = 41) [26], India (*n* = 79), Iran (*n* = 50), South Korea (*n* = 39), Brazil (*n* = 41) [27], Mexico (*n* = 19) [16], Colombia (*n* = 25), Sudan (*n* = 30) [28], Vanuatu (*n* = 21) [29], and Vietnam (*n* = 116) were included (Supplementary Material File S2: Table S1). Genetic polymorphism and the test of neutrality were analyzed for the *pvcsp* population with DnaSP ver 5.10.00 [21] and MEGA6 [22]. Polymorphic patterns of the N-terminal non-repeat region in global *pvcsp* was analyzed, and a logo plot for each population was constructed using the WebLogo program (https://weblogo.berkeley.edu/logo.cgi; accessed on 6 April 2022).

## 3. Results

### 3.1. Allelic Diversity of Vietnamese pvcsp

**Figure 1:**
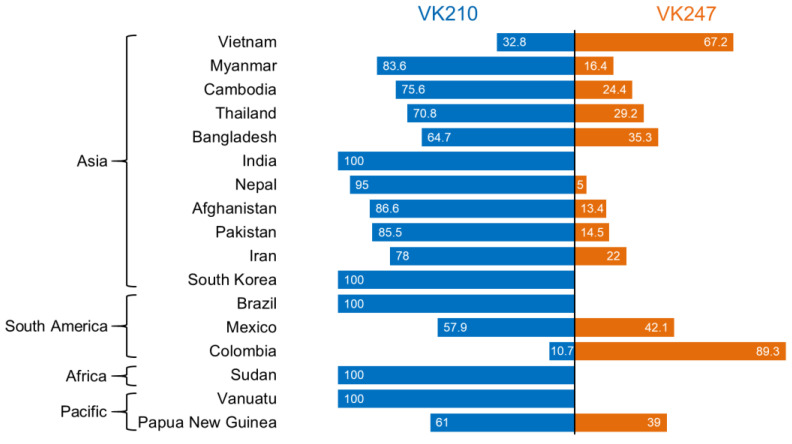
Allelic diversity of *pvcsp* in global *P. vivax* isolates. Global allelic diversity of *pvcsp* was comparatively analyzed. The bars represent percentage of *pvcsp* allelic types (VK210 and VK247) in each country. Information for each country’s VK210 and VK247 allelic types was obtained from the GenBank databank or previously published articles (Supplemental File S2: Table S1). Myanmar [17]; Cambodia [26]; Thailand [30]; Bangladesh [31]; India (GenBank); Nepal [32]; Afghanistan [33]; Iran (GenBank); South Korea (GenBank); Brazil [27]; Mexico [16]; Colombia; Sudan [28]; Vanuatu [29]; Papua New Guinea [34].

PCR amplification of *pvcsp* from 69 Vietnamese *P. vivax* isolates successfully yielded 116 *pvcsp* amplicons with different sizes ranging from 0.3 kb to 0.8 kb. Multi-clonal infections, presumed to have at least two different sizes of amplicons in an isolate, were detected in 43 isolates. The mean multiplicity of infection was 1.68. Among the 43 samples with multiclonal infections, 4 samples were multiple infections with different allele types of VK210 and VK247. Sequence analysis of these 116 *pvcsp* amplicons revealed only two allelic variants of Vietnamese *pvcsp*, VK210, and VK247, but not the *P. vivax*-like variant (Figure 1). The VK247 variants were predominant, accounting for 67.2% (*n* = 78). The VK210 variants accounted for the remaining 32.8% (*n* = 38). A comparative analysis of the global distribution of these two major *pvcsp* variants revealed that the proportions of these two major *pvcsp* variants differed by country. However, geographical relevance was not observed (Figure 1). The VK210 variants were predominant in most countries with different frequencies, whereas the VK247 variants were prevalent in Vietnam and Colombia.

### 3.2. Polymorphism of the N-Terminal Non-Repeat Region in Vietnamese pvcsp

**Figure 2:**
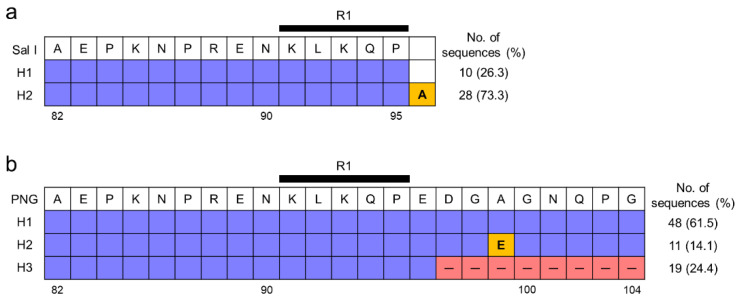
Polymorphic patterns of the N-terminal non-repeat region of Vietnamese *pvcsp*. (**a**) VK210 variants. Two different haplotypes were identified in 38 Vietnamese VK210 sequences. The blue box represents amino acid residues identical to the reference sequence of Sal I (GU339059). An alanine insertion is indicated by a yellow box. (**b**) VK247 variants. Three distinct haplotypes were detected in 78 Vietnamese VK247 sequences. The blue box represents residues identical to the reference sequence of PNG (M69059). The A99E substitution is marked by a yellow box. Dashes in red boxes represent deletions. RI means a KLKQP motif was involved in the sporozoite invasion of mosquito salivary gland and binding to hepatocytes before invasion [12]. The total number of sequences and frequency for each haplotype are listed on the right side.

**Figure 3:**
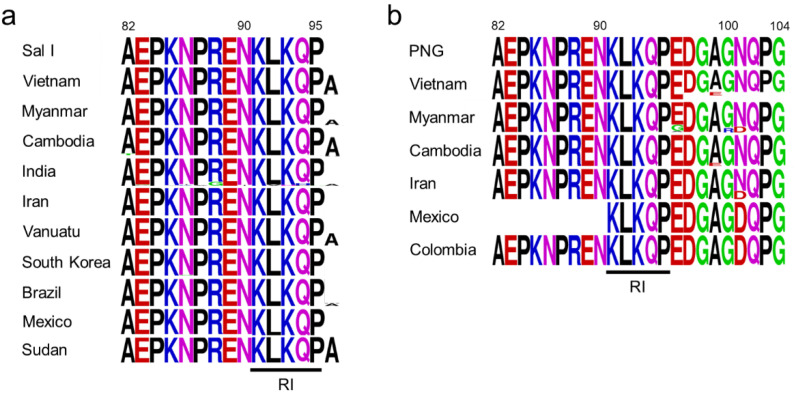
Polymorphism patterns of the N-terminal non-repeat region in global *pvcsp*. (**a**) Global VK210 variants. (**b**) Global VK247 variants. Different patterns of amino acid polymorphism by country or geographic continent were detected. Sal I, Salvador I (GU339059); PNG, Papua New Guinea (M69059). RI region was marked by an underline.

The N-terminal non-repeat region of Vietnamese *pvcsp* was highly conserved (Figure 2). The VK210 variants were divided into only two different haplotypes (Figure 2a). Haplotype 1 (H1) had identical amino acid sequences with the Sal I reference strain, while haplotype 2 (H2) had an alanine insertion at the end of the highly conserved R1 motif (^91^KLKQP^95^). Twenty-eight (73.7%) sequences belonged to H2, while the remaining ten (26.3%) sequences were classified into H1. For the VK247 variants, three distinct haplotypes, H1, H2, and H3, were detected (Figure 2b). H1, which had amino acid sequences identical to the PNG reference strain, was predominant with a frequency of 61.5%. H2 had a single amino acid substitution at position 99 (A99E). H3 differed from the two haplotypes in that it had an octapeptide deletion (^97^DGAGNQPG^104^) at the end of the N-terminal region. Polymorphic patterns of the N-terminal region of global *pvcsp* were also analyzed by combining Vietnamese *pvcsp* sequences and currently available *pvcsp* sequences in the GenBank database. As reported previously [17], the global *pvcsp* population displayed a low level of polymorphic characters in the N-terminal region. In the VK210 variants, insertion of an alanine at the end of the C-terminus was the most notable variation, albeit several rare amino acid substitutions were detected in the Indian VK210 variants (Figure 3a). Meanwhile, the global VK247 variants showed more polymorphisms than the VK210 variants (Figure 3b). Amino acid changes in the last nine amino acids of the C-terminal end were major factors causing polymorphic patterns. Particularly, N101D was the most remarkable amino acid substitution found in the VK247 variants from Colombia (100%), Mexico (100%), Iran (27.3%), and Myanmar (17.9%). Several amino acid changes, including E96G, A99E, and G100R, were also detected in some countries. Octapeptide deletion (^97^DGAGNQPG^104^) was found in only the VK247 variants from Vietnam and Myanmar. Despite these sequence variations and insertion/deletion in the global VK210 and VK247 variants, the R1 motif was well conserved in both variants of global *pvcsp*.

### 3.3. Polymorphic Pattern of the CRR in Vietnamese pvcsp

**Figure 4:**
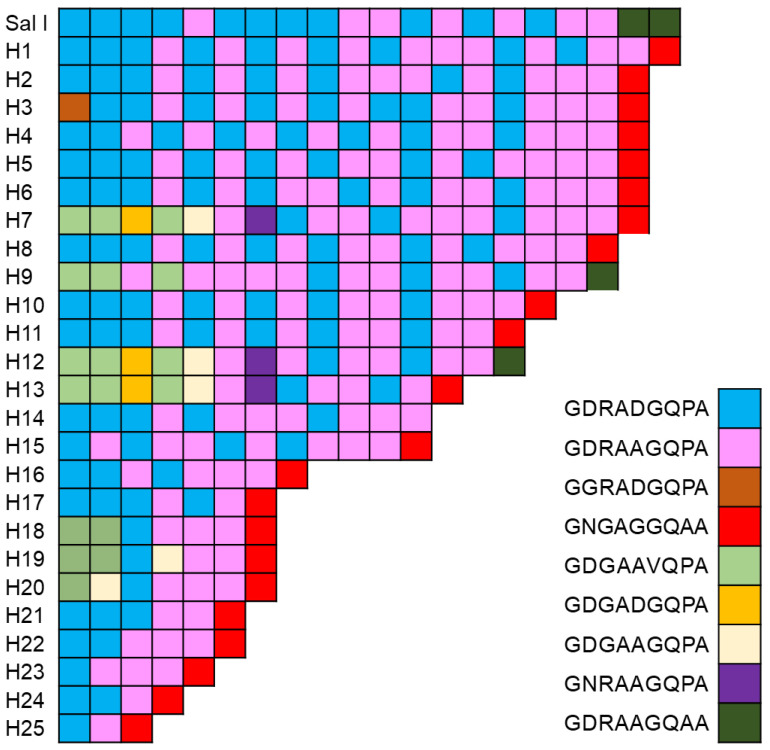
Polymorphic pattern of the central repeat region (CRR) of the Vietnamese VK210 variants. The CRR of the Vietnamese VK210 variants revealed polymorphic characters with 25 distinct haplotypes. A total of 9 different types of peptide repeat motifs (PRMs) were detected in the CRR of the Vietnamese VK210 variants. Differences in types, numbers, and combinations of PRMs produced polymorphisms of the CRR in the Vietnamese VK210 variants.

**Figure 5:**
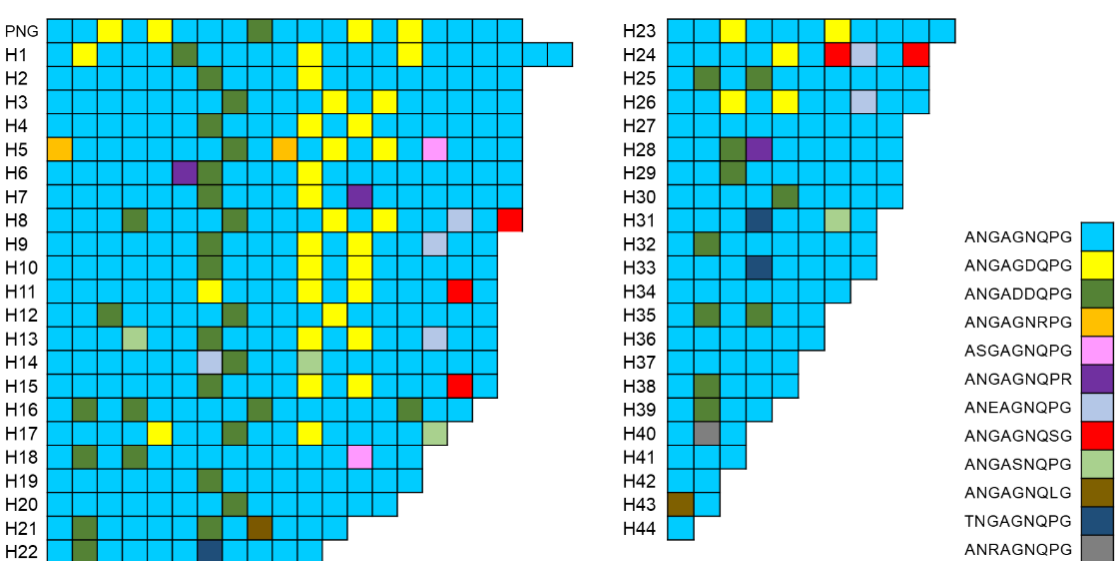
Polymorphic pattern of the central repeat region (CRR) of the Vietnamese VK247 variants. The CRR of the Vietnamese VK247 variants showed polymorphic characters with 44 distinct haplotypes. A total of 12 different types of peptide repeat motifs (PRMs) were identified in the CRR of the Vietnamese VK247 variants. Differences in types, numbers, and combinations of PRMs produced the polymorphism of the CRR in Vietnamese VK247 variants.

The CRR region of Vietnamese *pvcsp* displayed high levels of size in polymorphisms and sequence variations in both the VK210 and VK247 variants. In the VK210 variants, nine species of PRMs, including GDRADGQPA, GDRAAGQPA, GGRADGQPA, GNGAGGQAA, GDGAAVQPA, GDGADGQPA, GDGAAGQPA, GNRAAGQPA, and GDRAAGQAA, were basic building blocks constructing the CRR (Figure 4). Two PRMs, GDRADGQPA and GDRAAGQPA, were predominant. Nine different types of PRMs were arranged with varying copies and combinations in the CRRs, resulting in 25 distinct haplotypes (H1–H25) with size polymorphisms and different PRM constructs in the Vietnamese VK210 variants. The copy number of PRMs detected in the CRRs of the Vietnamese VK210 variants varied from 3 to 20. The most predominant haplotype was H8 (*n* = 8). Most haplotypes started with GDRADGQPA and terminated with GNGAGGQAA. The CRR of the Vietnamese VK247 variants displayed more complex patterns than that of VK210. A total of 12 distinct types of PRMs were detected. They constructed 44 distinguished haplotypes (H1–H44) with different copy numbers and combinations of PRMs (Figure 5). ANGAGNQPG and ANGADDQPG were major PRMs constructing the CRRs of the Vietnamese VK247 variants. The most predominant haplotype was H42 (*n* = 6). Most haplotypes started with ANGAGNQPG and ended with the same PRM. Only H5 and H43 started with different PRMs, which were ANGAGNRPG and ANGAGNQLG, respectively. Meanwhile, three haplotypes (H8, H17, and H24) were terminated with ANGAGNQSG or ANGASNQPG. The 8 PRMs, ANGAGNRPG, ASGAGNQPG, ANGAGNQPR, ANEAGNQPG, ANGASNQPG, ANGAGNQLG, TNGAGNQPG, and ANRAGNQPG, identified in the Vietnamese VK247 variants were novel ones that were not previously reported.

### 3.4. Size Polymorphism in the CRR of the Global pvcsp

**Figure 6:**
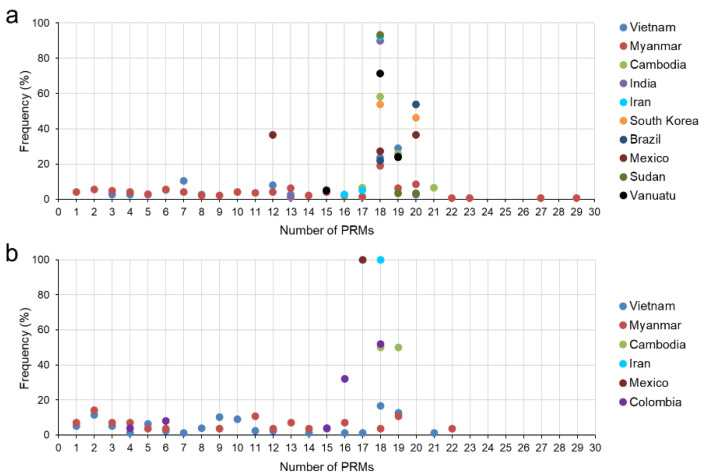
Length polymorphism of the central repeat region (CRR) caused by varying numbers of peptide repeat motifs (PRMs) in global *pvcsp*. (**a**) Global VK210 variants. (**b**) Global VK247 variants.

The CRR of the global *pvcsp* showed highly polymorphic patterns. A total of 98 types of PRMs were found in the global VK210 variants, some of which were uniquely identified in a particular country (Supplement File S3: Table S2). Different types of PRMs and different arrangements of PRMs generated polymorphic characters of the CRR in the global *pvcsp* population. A high degree of size polymorphisms was detected in the CRR of the global VK210 variants (Figure 6a). The range of size variations in the CRR differed by country, with the VK210 variants from Vietnam and Myanmar showing greater size polymorphisms in the CRR than those from other countries. For the VK247 variants, less diverse types of PRMs (18 types) were detected in the global VK247 variants (Supplement File S4: Table S3). Size polymorphisms were also observed in the CRR of the global VK247 variants, but they were less diverse than those in the VK210 variants (Figure 6b). Similar to the VK210 variants, greater size polymorphisms were also detected in the CRR of the Myanmar and Vietnamese VK247 variants than in other countries.

### 3.5. Polymorphism of the C-Terminal Non-Repeat Region in Vietnamese pvcsp

**Figure 7:**
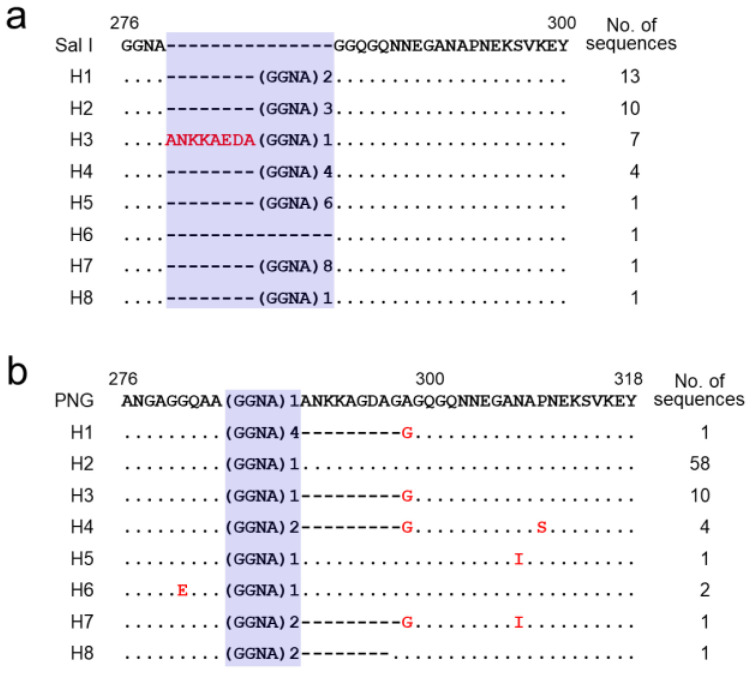
Polymorphic patterns of the C-terminal non-repeat region of Vietnamese *pvcsp*. (**a**) VK210 variants. A total of eight distinct haplotypes of the C-terminal non-repeat region were identified in 38 Vietnamese VK210 sequences. The dots represent residues identical to the reference sequence of Sal I (GU339059). The number of GGNA repeat motifs is presented. Dashes represent gaps to maximize the alignment. The number of sequences for each haplotype is listed on the right panel. (**b**) VK247 variants. Eight distinct haplotypes of the C-terminal non-repeat region was identified in 78 Vietnamese VK247 sequences. The dots represent residues identical to the reference sequence of PNG (M69059). The number of GGNA repeat motifs is presented. Amino acid changes at particular amino acid positions are indicated in red. Dashes represent gaps to maximize the alignment. The total number of sequences for each haplotype is listed on the right panel.

**Figure 8:**
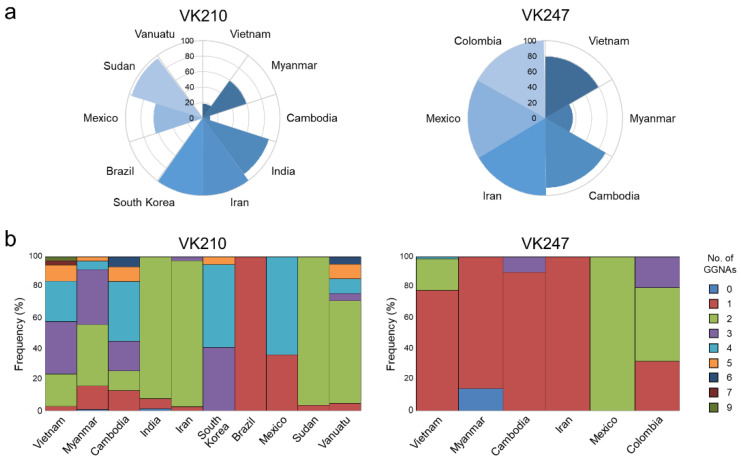
Frequencies of octapeptide insertion and GGNA motifs in the C-terminal non-repeat region of global *pvcsp*. (**a**) Frequency of octapeptide (ANKKAEDA in VK210 and ANKKAGDA in VK247) in the C-terminal non-repeat region of global VK210 and VK247 variants. (**b**) Frequency of number of GGNA repeat motifs in the C-terminal non-repeat region of global VK210 and VK247 variants. Different numbers of GGNA motifs repeated in the sequences.

The C-terminal non-repeat region of Vietnamese *pvcsp* showed limited variations. The C-terminal region of the VK210 variants revealed eight distinct haplotypes, H1–H8 (Figure 7a). No amino acid substitution was detected in any haplotypes. However, insertions of the ANKKAEDA octapeptide and different copy numbers (1 to 9) of the GGNA repeat motifs produced polymorphic characters in the region. H1 was the most predominant haplotype, followed by H2, H3, and H4. H3 had a unique ANKKAEDA octapeptide insertion. Different copy numbers of the GGNA motifs were found in all haplotypes except for H6. The C-terminal region of the Vietnamese VK247 variants also showed polymorphic characters resulting in 8 different haplotypes, H1–H8 (Figure 7b). H2, identical to the PNG reference sequence, was the most prevalent haplotype, followed by H3 and H4. Several amino acid changes were detected at four positions, namely G281E, A298G, N308I, and P310S. Different copy numbers (1 to 4) of the GGNA repeat motifs also contributed to polymorphic characters of the C-terminal region in the Vietnamese VK247 variants. Substantial levels of polymorphic characters were also detected in the C-terminal region of global *pvcsp*. The frequency of ANKKAEDA octapeptide insertion varied among the VK210 variants from different countries (Figure 8a). All VK210 variants from Iran and South Korea had this octapeptide insertion in the region. The frequency of VK210 variants with the octapeptide insertion differed by country: Sudan (96.7%); India (89.9%); Mexico (63.6%); Myanmar (59.4%); Vietnam (18.4%); and Cambodia (9.7%). Meanwhile, the VK210 variants from Brazil and Vanuatu did not harbor this insertion. For the VK247 variants, the ANKKAGDAG octapeptide was detected in all VK247 variants from Colombia, Iran, and Mexico (Figure 8a). Frequencies of the VK247 variants with the octapeptide insertion in Cambodia, Vietnam, and Myanmar were 90.0%, 78.2%, and 35.7%, respectively. The copy numbers of the GGNA motifs in the region among the global VK210 variants also differed by country (Figure 8b). Numerical variations of the GGNA repeat motif were greater in the VK210 variants from Vietnam, Myanmar, Cambodia, and Vanuatu than those from other countries. For the global VK247 variants, the copy number of the GGNA motifs found in the C-terminal region ranged from 0 to 4 (Figure 8b). The copy number of the GGNA repeat motifs in global VK247 also differed by country, although such differences were less diverse than those in the VK210 variants.

### 3.6. Nucleotide Diversity and Natural Selection in N- and C-Terminal Non-Repeat Regions of Vietnamese and Global pvcsp VK210 Variants

The nucleotide diversity and natural selection of N- and C-terminal non-repeat regions in Vietnamese and global *pvcsp* were comparatively analyzed. For the N-terminal region of Vietnamese VK210, the average number of nucleotide differences (*K*), overall haplotype diversity (Hd), and nucleotide diversity (π) were 0.102, 0.102 ± 0.065, and 0.0024 ± 0.0016, respectively (Table 1). These values for the C-terminal region of Vietnamese VK210 were 0.713, 0.383 ± 0.081 and 0.0095 ± 0.0020, respectively. The dN–dS values for both N- and C-terminal regions were negative, suggesting that these regions were under negative natural selection. Tajima’s D value for the N-terminal region was negative (–0.8255), whereas this value for the C-terminal region was positive (0.9518). However, these values were not statistically significant (*p* > 0.1). Fu and Li’s D and F values were positive for both the N- and C-terminal regions of Vietnamese VK210. The N-terminal region of Vietnamese VK210 showed similar or slightly higher values of *K*, Hd, and π than that of the global VK210 variants, except for the Indian VK210 variants. The N-terminal region of the VK210 variants showed different trends in natural selection by country. The dN–dS values were negative for the VK210 variants from Vietnam, India, and Brazil, whereas such values for the VK210 variants originating from Myanmar, Cambodia, and South Korea were positive, suggesting a positive natural selection. For the VK210 variants from Iran, Mexico, Sudan, and Vanuatu, these values were neutral (0) as only one haplotype was identified from each country. Tajima’s D values for the N-terminal region of the VK210 variants from Vietnam, Myanmar, Cambodia, India, South Korea, and Brazil were negative, suggesting a purifying selection in the region. The C-terminal region of the global VK210 variants also showed nucleotide diversity and patterns of natural selection. The Vietnamese VK210 variants displayed the greatest nucleotide diversity. This might be attributed to the octapeptide deletion (^97^DGAGNQPG^104^) observed in the Vietnamese VK210 variants. Meanwhile, no nucleotide diversity (π = 0) was found in the VK210 variants from Cambodia, Iran, Brazil, Mexico, and Vanuatu. The C-terminal region of global VK210 was also under neutral or purifying selection considering that its Tajima’s D values were 0 or negative. However, the C-terminal region of Vietnamese VK210 had a positive Tajima’s D value, suggesting a balancing selection.

### 3.7. Nucleotide Diversity and Natural Selection in N- and C-Terminal Non-Repeat Regions of Vietnamese and Global pvcsp VK247 Variants

Similar to the VK210 variants, substantial levels of nucleotide diversity and natural selection were also found in the Vietnamese VK247 variants (Table 2). The K, Hd, and π values for the N-terminal region of the Vietnamese VK247 variants were 0.370, 0.370 ± 0.050, and 0.0082 ± 0.0011, respectively. For the C-terminal region, these values were 1.357, 0.531 ± 0.063, and 0.0133 ± 0.0019, respectively. The dN–dS value was positive (0.0110) for the N-terminal region but negative for the C-terminal region (−0.0290), suggesting that opposite natural selections individually affected these two termini. The same phenomenon was also detected for the VK247 variants from Myanmar and Cambodia. Tajima’s D value, as well as Fu and Li’s D and F values of the Vietnamese N-terminal region of VK247 were all positive, implying that this region was under a balancing selection. The N-terminal region of the Iranian VK247 variants also showed a positive result for Tajima’s D value and Fu and Li’s D and F values. However, these values were negative for the N-terminal region of the VK247 variants from Cambodia and Myanmar. The C-terminal region of the global VK247 variants also displayed different patterns of natural selection. The dN–dS values for the C-terminal region of the VK247 variants from Vietnam, Myanmar, and Cambodia were negative, but they were positive for the VK247 variants from Colombia. Tajima’s D values for the C-terminal region of the VK247 variants from Vietnam, Myanmar, Cambodia, and Colombia were negative, suggesting that this region was under a purifying selection.

## 4. Discussion

The malaria burden in Vietnam has remarkably reduced in the recent years. However, the Central Highlands remains a major malaria transmission area [20]. The increasing prevalence and high genetic heterogenicity of *P. vivax* in the Central Highlands is also becoming a major concern in current efforts to control and eliminate malaria in the endemic areas [17]. Understanding the genetic structure of *Plasmodium* populations in the endemic areas will provide fundamental data for evaluating and improving efforts to control and eliminate malaria in Vietnam. However, limited information on the genetic make-up or structure of Vietnamese malaria parasites, both for *P. falciparum* and *P. vivax*, is currently available [35,36,37,38,39,40]. In this study, the genetic diversity and natural selection of *pvcsp* in Vietnamese *P. vivax* isolates collected in Central Highlands, Vietnam were analyzed to understand the genetic structure of the parasite and to provide expanded information in designing a vivax malaria vaccine based on PvCSP, a leading vaccine candidate.

Only two allelic variants of *pvcsp*, namely VK210 and VK247, were identified in the Vietnamese *pvcsp* population analyzed in this study. Interestingly, the VK247 allele was a dominant allele accounting for 67% in Vietnam. This does not coincide with the general trend that VK210 alleles are prevalent in other neighboring Southeast Asian countries, including Cambodia, Myanmar, and Thailand [17,26,41]. The reason for this discordant distribution of *pvcsp* alleles in Vietnam is not apparent. The population of mosquito vectors has been proposed as a potential factor affecting the distribution of *pvcsp* alleles in endemic areas [42]. In Southeast Asia, the *Anopheline* fauna showed a greater species diversity than that of other places in the world [43]. At least 19 *Anopheles* species have been identified as vectors transmitting malaria in Asia-Pacific region [44]. In Vietnam, a total of 61 *Anopheles* species have been recorded and 16 of them, such as *An. Dirus, An. Minimus, An. Harrisoni,* and *An. Epiroticus,* have been recognized as vector species transmitting malaria [45,46]. Further study on the differences in infectivity of the VK210 and VK247 variants in the particular species of *Anopheles* mosquitoes in Vietnam is necessary to understand the differences in the adaptability of each allelic variant to different mosquito vectors that affect the prevalence of VK247 in Vietnam.

Consistent with previous studies on the genetic diversity of global *pvcsp* [17,26,47,48], the Vietnamese VK210 and VK247 variants also showed low genetic polymorphisms in N- and C-terminal regions. However, several amino acid changes and size polymorphisms were also detected in these regions. The most notable amino acid change in Vietnamese VK210 was an alanine insertion at the end of the RI motif in the N-terminal region. This mutation was previously detected in other global populations with a different prevalence [17], although geographical relevance was not identified. The C-terminal region of Vietnamese VK210 also showed polymorphic characters caused by octapeptide insertion and the GGNA repeat motifs repeating with different copy numbers. The frequency of octapeptide insertion was not high in Vietnamese VK210 variants compared to those in other countries where this insertion was detected. However, the variation in copy numbers of the GGNA repeat motifs was greater in the Vietnamese VK210 variants than in the VK210 variants from other countries. Interestingly, copy number variations of these motifs were also higher in VK210 variants from Southeast Asian countries, including Myanmar and Cambodia, than in those from other continents. The N-terminal region of Vietnamese VK247 variants showed polymorphic patterns due to an amino acid substitution (N101D) and octapeptide deletion (^97^DGAGNQPG^104^). Similar to other previously reported VK247 variants [17], few amino acid changes and GGNA repeats also caused genetic polymorphism in the C-terminal region of Vietnamese VK247 variants. However, the copy number variation of the motifs was much lower in global VK247 than in global VK210. Despite these sequence variations and the insertion/deletion in global VK210 and VK247 variants, the N- and C-terminal regions of global *pvcsp* were relatively well-conserved without a significant regional or continental difference in the global *pvcsp* population. Notably, the R1 motif acting in the invasion of the malaria parasite into host cells [49] was tightly conserved in both the VK210 and VK247 variants of global *pvcsp*, suggesting that this motif might be an attractive target for designing a PvCSP-based vaccine [17]. Analyses of the N- and C-terminal regions of the Vietnamese VK210 and VK247 variants revealed that these regions were under natural selection. The dN–dS values for the N- and C-terminal regions of VK210 were negative (–0.0120) and positive (0.0110), respectively, suggesting that these two regions were under opposite selection pressure. Meanwhile, these values for both the N- and C-terminal regions of the VK247 variants were positive, suggesting that balancing selection might act in these two regions. The dN–dS values for the global VK210 and VK247 variants differed by country, indicating different natural selection pressure in global VK210 and VK247. Tajima’s D value, as well as Fu and Li’s D and F values, also revealed complex patterns of natural selection in the N- and C-terminal regions of global VK210 and VK247, which were unique in each country. Interestingly, the C-terminal region of Vietnamese VK210 and the N-terminal region of Vietnamese VK247 showed positive values of Tajima’s D value and Fu and Li’s D and F values, which were not matched with those from other countries. Balancing selection is likely to be the major driving force that induces genetic diversity in the two regions of Vietnamese VK210 and VK247.

The CRR of both the Vietnamese VK210 and VK247 variants showed high levels of genetic polymorphisms. Different copy numbers and arrangements of diverse species of PRMs induced genetic diversity of the CRR in Vietnamese *pvscp*. Similar patterns in the genetic complexity of the CRR were also identified in the global VK210 and VK247 variants. Up to now, highly various PRMs, which probably originated from a common ancestor [50], have been identified in global *pvcsp*. New point mutations in PRMs might generate novel PRMs, which can further increase the genetic diversity of the CRR and contribute to the appearance of unique haplotypes in global *pvcsp* [17]. A recombination during meiosis or an intrahelical strand slippage during mitosis [51] might also cause genetic diversity of the CRR. These emphasize the importance of continuous molecular surveillance of the genetic polymorphism of global *pvcsp*. Due to high levels of size polymorphisms in the CRR caused by varying numbers and arrangements of diverse PRMs, we could not conduct a direct analysis of the natural selection of the CRR in Vietnamese and global *pvcsp*. However, evidence supporting natural selection in the CRR has been previously reported [26,50,52].

The limitation of this study was the small size of samples. Considering malaria is mainly prevalent in the Central Highlands, Vietnam, studies with larger numbers of *P. vivax* isolates collected at different geographical sites in the endemic areas are necessary to gain an in-depth knowledge on the genetic structure of the parasite. Further analyses of genetic make-up of other polymorphic markers are also necessary.

## 5. Conclusions

PvCSP is one of the most intensively studied vaccine candidate antigens. However, the genetic diversity of *pvcsp* in the global *P. vivax* population can be a hurdle when developing a universal vaccine based on PvCSP. Like other global *pvcsp*, Vietnamese *pvcsp* also shows polymorphic characters throughout the gene, which is attributed to amino acid changes, insertions/deletions, and varying numbers of repeating PRMs. However, the Vietnamese *pvcsp* population displays a different evolutionary force from global *pvcsp*, especially with the neighboring Southeast Asian *pvcsp* population. The VK247 variants were prevalent. Substantial levels of genetic diversity and natural selection detected in Vietnamese *pvcsp* suggest that an adequate *P. vivax* population size remains to generate or maintain the genetic diversity of the parasite in Vietnam, despite a recent remarkable decline in malaria incidences. Moreover, substantial levels of multiplicity of infection and potential asymptomatic infections in the Central Highlands [20] have emphasized the importance of the continuous monitoring of the genetic diversity of *P. vivax* in the endemic area. The results of this study could expand our knowledge of the genetic structure of the Vietnamese *P. vivax* population and the global *pvcsp* population. Genetic polymorphic characters of global *pvcsp* also suggests that further analysis of genetic diversity in global *pvcsp* is essential to provide fundamental information for the development of an effective vaccine based on this antigen.

## Figures and Tables

**Table 1:** Nucleotide diversity and tests of neutrality in the N- and C-terminal non–repeat regions of global VK210 variants.

Region	Country	*n*	*K*	S	H	Hd ± SD	π ± SD	dN–dS	Tajima’s D*^p^* ^value^	Fu & Li’s D*^p^* ^value^	Fu & Li’s F*^p^* ^value^
N-terminal	Vietnam	38	0.102	1	2	0.102 ± 0.065	0.0024 ± 0.0016	−0.0120	−0.8255^a^	0.5688^a^	0.2028^a^
	Myanmar^#^	143	0.098	5	7	0.096 ± 0.034	0.0024 ± 0.0009	0.0008	−1.9359^c^	−3.9086^b^	−3.8409^b^
	Cambodia^#^	31	0.125	1	2	0.125 ± 0.077	0.0030 ± 0.0018	0.0039	−0.7737^a^	0.5907^a^	0.2450^a^
	India^#^	79	1.972	23	28	0.661 ± 0.063	0.0470 ± 0.0074	−0.0909	−2.0261^c^	−1.3952^a^	−1.9514^e^
	Iran^#^	39	0	0	1	0	0	0	0	0	0
	South Korea^#^	39	0.051	1	2	0.051 ± 0.048	0.0012 ± 0.0011	0.0016	−1.1264^a^	−1.7662^a^	−1.8293^a^
	Brazil^#^	41	0.049	1	2	0.049 ± 0.046	0.0012 ± 0.0011	−0.0059	−1.1219^a^	−1.7816^a^	−1.8406^a^
	Mexico^#^	11	0	0	1	0	0	0	0	0	0
	Sudan^#^	30	0	0	1	0	0	0	0	0	0
	Vanuatu^#^	21	0	0	1	0	0	0	0	0	0
C-terminal	Vietnam	38	0.713	2	3	0.383 ± 0.081	0.0095 ± 0.0020	−0.0450	0.9518^a^	0.7771^a^	0.9581^a^
	Myanmar^#^	143	0.209	10	11	0.186 ± 0.044	0.0035 ± 0.0009	−0.0035	−2.2251^d^	−3.5089^b^	−3.6329^b^
	Cambodia^#^	31	0	0	1	0	0	0	0	0	0
	India^#^	79	0.276	8	6	0.123 ± 0.051	0.0043 ± 0.0020	−0.0149	−2.1953^d^	−4.4419^b^	−4.3524^b^
	Iran^#^	39	0	0	1	0	0	0	0	0	0
	South Korea^#^	39	0.103	2	3	0.101 ± 0.065	0.0008 ± 0.0005	−0.0037	−1.4889^a^	−2.4148^e^	−2.4864^e^
	Brazil^#^	41	0	0	1	0	0	0	0	0	0
	Mexico^#^	11	0	0	1	0	0	0	0	0	0
	Sudan^#^	30	0.067	1	2	0.067 ± 0.061	0.0009 ± 0.0008	−0.0041	−1.1470^a^	−1.6821^a^	−1.7655^a^
	Vanuatu^#^	21	0	0	1	0	0	0	0	0	0

*n* = number of analyzed sequences; *K* = average number of nucleotide differences; S = number of segregating sites; H = number of haplotypes; Hd = haplotype diversity; π = observed average pairwise nucleotide diversity; SD = standard deviation; dN = rate of non–synonymous mutations; dS = rate of synonymous mutations. ^a^ *p* > 0.1; ^b^ *p* < 0.02; ^c^ *p* < 0.05; ^d^ *p* < 0.01; ^e^ 0.05 < *p* < 0.1. ^#^ Cited from [17].

**Table 2:** Nucleotide diversity and tests of neutrality in the N- and C-terminal regions of global VK247 variants.

Region	Country	*n*	*K*	S	H	Hd ± SD	π ± SD	dN–dS	Tajima’s D*^p^* ^Value^	Fu & Li’s D*^p^* ^Value^	Fu & Li’s F*^p^* ^Value^
N-terminal	Vietnam	79	0.370	1	2	0.370 ± 0.050	0.0082 ± 0.0011	0.0110	0.9975^a^	0.5078^a^	0.7588^a^
	Myanmar^#^	28	0.405	1	3	0.405 ± 0.094	0.0090 ± 0.0021	0.0117	–0.4445^a^	−0.7114^a^	−0.7369^a^
	Cambodia^#^	10	0.200	1	2	0.200 ± 0.154	0.0029 ± 0.0022	0.0038	−1.1117^a^	−1.2434^a^	−1.3467^a^
	Iran^#^	11	1.309	3	2	0.436 ± 0.133	0.0190 ± 0.0058	−0.0543	0.9518^a^	1.1271^a^	1.2185^a^
	Mexico^#^	8	0	0	1	0	0	0	0	0	0
	Colombia^#^	25	0	0	1	0	0	0	0	0	0
C-terminal	Vietnam	79	1.357	8	9	0.531 ± 0.063	0.0133 ± 0.0019	−0.0290	−0.4083^a^	1.2744^a^	0.8376^a^
	Myanmar^#^	28	0.495	6	6	0.331 ± 0.114	0.0068 ± 0.0027	−0.0150	−1.9719^b^	−2.5946^b^	−2.8039^b^
	Cambodia^#^	10	0.400	2	2	0.200 ± 0.154	0.0039 ± 0.0030	−0.0172	−1.4009^a^	−1.5866^a^	−1.7190^a^
	Iran^#^	11	0	0	1	0	0	0	0	0	0
	Mexico^#^	8	0	0	1	0	0	0	0	0	0
	Colombia^#^	25	0.500	2	3	0.440 ± 0.095	0.0039 ± 0.0010	0.0017	−0.1215^a^	−0.6754^a^	−0.6012^a^

*n* = number of analyzed sequences; *K* = average number of nucleotide differences; S = number of segregating sites; H = number of haplotypes; Hd = haplotype diversity; π = observed average pairwise nucleotide diversity; SD = standard deviation; dN = rate of non-synonymous mutations; dS = rate of synonymous mutations. ^a^ *p* > 0.1; ^b^ *p* < 0.05. ^#^ Cited from [17].

## Data Availability

The data supporting the conclusions of this article are provided within the article. The original datasets analyzed in this study are available from the corresponding author (B.-K.N.) upon request. The nucleotide sequences reported in this study have been deposited in the GenBank database under the accession numbers MW382969–MW383084.

## Supplementary Materials

The following supporting information can be downloaded at: https://www.mdpi.com/article/10.3390/pathogens11101158/s1, Supplement File S1: Figure S1. Map of blood collection areas, the Central Highlands, Vietnam. Finger-prick blood samples were collected from *P. vivax* infected patients in the Central Highlands, Vietnam; Supplement File S2: Table S1. List of global *pvcsp* sequences analyzed in this study; Supplement File S3: Table S2. List of peptide repeat motifs (PRMs) identified in the CRR of global VK210 variants; Supplement File S4: Table S3. List of peptide repeat motifs (PRMs) identified in the CRR of global VK247 variants.
