# Supplementary material for: Genetic Diversity of Circumsporozoite Surface Protein of Plasmodium vivax from the Central Highlands, Vietnam
Source: Pathogens. 2022 Oct 7;11(10):1158. doi: 10.3390/pathogens11101158 (PMC9611680; doi:10.3390/pathogens11101158)
Supplement: Supplementary file 1 [file pathogens-11-01158-s001.zip › Vo TC et al._Supplement file S2_Table S1.pdf]

Table S1. List of global *pvcsp* sequences analysed in this study

Variant type	Country	Number of sequences	GenBank accession numbers
VK210	Myanmar	143	MN821829–MN821971
	Cambodia	31	JX461243, JX461245– JX461248, JX461250, JX461252, JX461254–JX461258, JX461260–JX461267, JX461269, JX461271–JX461274, JX461277–JX461279, JX461282–JX461284
	India	79	FJ491064–FJ491141
	Iran	39	KT588159–KT588161, KT588164–KT588172, KT588174, KT588175, KT588177, KT588178, KT588180, KT588182, KT588184, KT588186, KT588188–KT588195, KT588197–KT588205, KT588207, KT588208
	South Korea	39	DQ859734–DQ859772
	Brazil	41	DQ978649–DQ978689
	Mexico	11	JQ511263–JQ511269, JQ511271, JQ511273, JQ511276, JQ511280
	Sudan	30	KP162217–KP162246
	Vanuatu	21	AB539022, AB539023, AB539025–AB539029, AB539031–AB539038, AB539040–AB539045
VK247	Myanmar	28	MN821972–MN821999
	Cambodia	10	JX461244, JX461249, JX461251, JX461259, JX461268, JX461275, JX461276, JX461280, JX461281, JX461285
	Iran	11	KT588162, KT588163, KT588173, KT588176, KT588179, KT588181, KT588183, KT588185, KT588187, KT588196, KT588206
	Mexico	8	JQ511270, JQ511272, JQ511274, JQ511275, JQ511277–JQ511279, JQ511281
	Colombia	25	GU339060–GU339071, GU339073–GU339084, GU339086
