# Supplementary material for: Genetic Diversity of Circumsporozoite Surface Protein of Plasmodium vivax from the Central Highlands, Vietnam
Source: Pathogens. 2022 Oct 7;11(10):1158. doi: 10.3390/pathogens11101158 (PMC9611680; doi:10.3390/pathogens11101158)
Supplement: Supplementary file 1 [file pathogens-11-01158-s001.zip › Vo TC et al._Supplement file S3_Table S2.pdf]

Table S2. List of peptide repeat motifs (PRMs) identified in the CRR of global VK210 variants

(■ = cell shaded green in the original table)

No.	PRM	Country									
		Vietnam	Myanmar	Cambodia	India	Iran	South Korea	Brazil	Mexico	Sudan	Vanuatu
1	**GDRADGQPA**	■	■	■	■	■	■	■	■	■	■
2	**GDRAAGQPA**	■	■	■	■	■	■	■	■	■	■
3	**GGRADGQPA**	■	■		■						
4	**GNGAGGQAA**	■	■	■	■	■	■	■	■	■	■
5	**GDGAAVQPA**	■	■	■							
6	**GDGADGQPA**	■	■	■							
7	**GDGAAGQPA**	■	■	■			■				
8	**GNRAAGQPA**	■	■								
9	**GDRAAGQAA**	■	■				■	■	■		■
10	**GNGAGGQPA**		■				■				
11	**GDRADGQAA**		■				■				
12	**GNRADGQPA**		■					■			
13	**GDSADGQPA**		■				■				
14	**GDRVAGQPA**		■								
15	**GVGAAGQPA**		■								
16	**GDRAHGQPA**		■								
17	**GDRADGKPA**		■								
18	**EDRADGQPA**		■		■						
19	**GDKADGQPA**		■								
20	**GDRADRQPA**		■								
21	**GDGAGGQAA**		■								
22	**GDRAAEQPA**		■								
23	**GDRVAGQPA**		■								
24	**GVGAAGQPA**		■								
25	**GDRAHGQPA**		■								
26	**GGRADGQPA**		■								
27	**GDRADGKPA**		■								
28	**EDRADGQPA**		■								
29	**GDKADGQPA**		■								
30	**GDRADRQPA**		■								
31	**GDGAGGQAA**		■								
32	**GDRAAEQPA**		■								
33	**EDRAAGQPA**		■								

34	**GDKAAGQPA**										
35	**GGRAAGQPA**										
36	**GDRAAGLPA**										
37	**GDRADVQPA**										
38	**GDRADGQPV**										
39	**GDRADGLPA**										
40	**GDRADGQPT**										
41	**GDRAARQPA**										
42	**GDRAAGRPA**										
43	**GDGAGGQPA**										
44	**GDSAAGQPA**										
45	**GDRAAGQSA**										
46	**SDRADGRPA**										
47	**GDRAAGQPT**										
48	**GDRAYGQPA**										
49	**GDRAVGQPA**										
50	**SDRAAGQPA**										
51	**RDRADGQPA**										
52	**GDRASGQPA**										
53	**GGRADGRPA**										
54	**GDRADQQPA**										
55	**GDRADGPPA**										
56	**GNGADGQPA**										
57	**GDRTAGQPA**										
58	**GARADGQPA**										
59	**GDRAADHPA**										
60	**GDRAAGHPA**										
61	**GEGADGQPA**										
62	**GDRQVGQPA**										
63	**GIRADGQPP**										
64	**GVRAAGHPA**										
65	**GDRADGHPA**										
66	**GDGADGHPA**										
67	**GVRAAGQPA**										
68	**GDRAVGHPA**										
69	**GDRAAVQPA**										
70	**GDRGAGQPA**										
71	**GDRGVGHPA**										

72	**GDRAAEHPA**										
73	**GDRAAAHPA**										
74	**GDRADAHPA**										
75	**GDRAAGHPS**										
76	**GDRAVAQPA**										
77	**GDRAADQPA**										
78	**GDRAVVQPA**										
79	**GDRAVEQPA**										
80	**GDRAGDHPA**										
81	**GDRGDGQPA**										
82	**GNGAGGQAP**										
83	**GDREDGQPA**										
84	**ETRADGQPA**										
85	**GDRADEQPA**										
86	**GDGAVGQPA**										
87	**GGGPVGQPA**										
88	**GARADEQPA**										
89	**GDRAADKPA**										
90	**GDRAEGQPA**										
91	**ADRGDGQPA**										
92	**ADRAAGQPA**										
93	**ADGAAGQPA**										
94	**ADRADGQPA**										
95	**DDGAAGQPA**										
96	**GDRAAGQPV**										
97	**DDRAAGQPA**										
98	**VDRADGQPA**										
