# Supplementary material for: Genetic Diversity of Circumsporozoite Surface Protein of Plasmodium vivax from the Central Highlands, Vietnam
Source: Pathogens. 2022 Oct 7;11(10):1158. doi: 10.3390/pathogens11101158 (PMC9611680; doi:10.3390/pathogens11101158)
Supplement: Supplementary file 1 [file pathogens-11-01158-s001.zip › Vo TC et al._Supplement file S4_Table S3.pdf]

Table S3. List of peptide repeat motifs (PRMs) identified in the CRR of global VK247 variants

No.	PRM	Country					
		Vietnam	Myanmar	Cambodia	Iran	Mexico	Colombia
1	**ANGAGNQPG**						
2	**ANGAGDQPG**						
3	**ANGADDQPG**						
4	**ANGAGNRPG**						
5	**ASGAGNQPG**						
6	**ANGAGNQPR**						
7	**ANEAGNQPG**						
8	**ANGAGNQSG**						
9	**ANGASNQPG**						
10	**ANGAGNQLG**						
11	**TNGAGNQPG**						
12	**ANRAGNQPG**						
13	**AYGAGNQPG**						
14	**VNGAGNQPG**						
15	**ANGVGNQPG**						
16	**ANGAGGQPG**						
17	**ANGAGKQPG**						
18	**ANGADNQPG**						
