# Supplementary figures and images for: Genetic Diversity of Circumsporozoite Surface Protein of Plasmodium vivax from the Central Highlands, Vietnam
Source: Pathogens. 2022 Oct 7;11(10):1158. doi: 10.3390/pathogens11101158 (PMC9611680; doi:10.3390/pathogens11101158)

## Slide 1
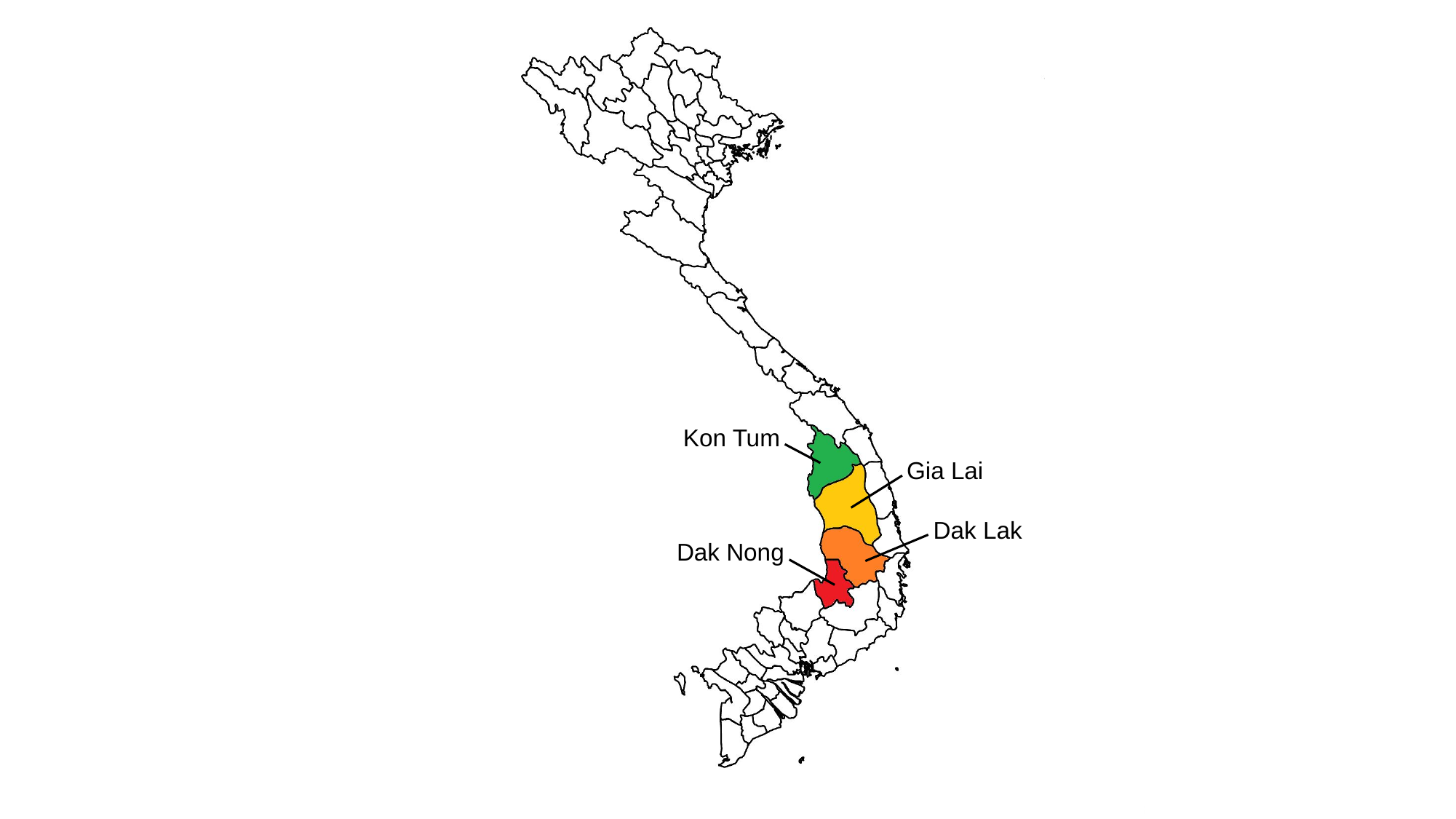

Kon Tum
Gia Lai
Dak Lak
Dak Nong

Supplement: Supplementary file 1 [file pathogens-11-01158-s001.zip › Vo TC et al._Supplement file S1_Figure S1.pptx]
